# Trimethylamine, a gut bacteria metabolite and air pollutant, increases blood pressure and markers of kidney damage including proteinuria and KIM-1 in rats

**DOI:** 10.1186/s12967-022-03687-y

**Published:** 2022-10-15

**Authors:** Klaudia M. Maksymiuk, Mateusz Szudzik, Marta Gawryś-Kopczyńska, Maksymilian Onyszkiewicz, Emilia Samborowska, Izabella Mogilnicka, Marcin Ufnal

**Affiliations:** 1 Department of Experimental Physiology and Pathophysiology, Laboratory of the Centre for Preclinical Research, Medical University of Warsaw, 02-091 Warsaw, Poland; 2 Spectrometry Laboratory, Institute of Biochemistry and Biophysics, Polish Academy of Sciences, Warsaw, Poland

**Keywords:** Trimethylamine, Trimethylamine oxide, Chronic kidney disease, Hypertension, Proteinuria, Kidney damage

## Abstract

**Background:**

Trimethylamine oxide (TMAO) is a biomarker in cardiovascular and renal diseases. TMAO originates from the oxidation of trimethylamine (TMA), a product of gut microbiota and manufacturing industries-derived pollutant, by flavin monooxygenases (FMOs). The effect of chronic exposure to TMA on cardiovascular and renal systems is undetermined.

**Methods:**

Metabolic, hemodynamic, echocardiographic, biochemical and histopathological evaluations were performed in 12-week-old male SPRD rats receiving water (controls) or TMA (200 or 500 µM/day) in water for 18 weeks. TMA and TMAO levels, the expression of FMOs and renin-angiotensin system (RAS) genes were evaluated in various tissues.

**Results:**

In comparison to controls, rats receiving high dose of TMA had significantly increased arterial systolic blood pressure (126.3 ± 11.4 vs 151.2 ± 19.9 mmHg; P = 0.01), urine protein to creatinine ratio (1.6 (1.5; 2.8) vs 3.4 (3.3; 4.2); P = 0.01), urine KIM-1 levels (2338.3 ± 732.0 vs. 3519.0 ± 953.0 pg/mL; P = 0.01), and hypertrophy of the tunica media of arteries and arterioles (36.61 ± 0.15 vs 45.05 ± 2.90 µm, P = 0.001 and 18.44 ± 0.62 vs 23.79 ± 2.60 µm, P = 0.006; respectively). Mild degeneration of renal bodies with glomerulosclerosis was also observed. There was no significant difference between the three groups in body weight, water-electrolyte balance, echocardiographic parameters and RAS expression. TMA groups had marginally increased 24 h TMA urine excretion, whereas serum levels and 24 h TMAO urine excretion were increased up to 24-fold, and significantly increased TMAO levels in the liver, kidneys and heart. TMA groups had lower FMOs expression in the kidneys.

**Conclusions:**

Chronic exposure to TMA increases blood pressure and increases markers of kidney damage, including proteinuria and KIM-1. TMA is rapidly oxidized to TMAO in rats, which may limit the toxic effects of TMA on other organs.

**Supplementary Information:**

The online version contains supplementary material available at 10.1186/s12967-022-03687-y.

## Background

The reciprocal interaction between the cardiovascular and renal systems is well established [1]. Furthermore, heart failure often coexists with renal failure, which is defined as a cardiorenal syndrome (CRS). There are several types of CRS, which are categorized by their primary cause (heart or kidney dysfunction) and duration (chronic or acute). Other comorbidities may also be involved in the development of CRS including diabetes mellitus or sepsis [2].

Recently, the association between CRS and gut microbiota has been intensively investigated [3–6]. For instance, it has been postulated that trimethylamine oxide (TMAO), one of the gut microbiota-derived metabolites, may be involved in the pathogenesis of cardiovascular and kidney diseases. Several studies have shown that patients with chronic kidney disease (CKD) have elevated plasma and/or urine TMAO concentration [7–10]. Besides, patients with end-stage renal failure show increased plasma TMAO levels, which decreases after hemodialysis [11]. Tang et al. found that TMAO contributes to progressive kidney dysfunction and renal fibrosis [10]. Finally, a positive correlation between high plasma TMAO and atherosclerosis [12–14], heart failure [15, 16] and hypertension [17, 18] was reported, and a causal relation between TMAO and cardiovascular disease (CVD) has been proposed. However, other studies suggest beneficial effect of TMAO in CVD [19–21]. Therefore, currently TMAO is considered a biomarker in CVD and renal diseases, but the causative role of TMAO in CVD is open to debate and may depend on TMAO concentration and animal species.

Interestingly, our previous studies found that trimethylamine (TMA), but not TMAO, exerts toxic effects on the cardiovascular system. Specifically, TMA increased blood pressure in anesthetized rats during the intravenous administration and exerted cytotoxic effects in *in vitro* studies [22–24].

Plasma TMAO originates from TMA, a gut bacteria product of choline and carnitine [25]. Other origins of TMA in the human body are also possible. First, TMA is manufactured on the order of thousands of tons worldwide. It is used in the production of plastics, disinfectants, insect attractants, intense sweeteners, seafood flavor, vitamin B4 and many other compounds. Second, TMA is an air pollutant [26]. TMA oxidation occurs mainly in the liver by the action of flavin monooxygenase 3 (FMO3) [27]. Both TMA and TMAO are removed from the body by the kidneys [25], although TMA was also detected in exhaled air in patients with end-stage renal disease [28]. The impact of TMA on human health is poorly determined; however, some studies suggested toxic effects of TMA [28–31].

To the best of our knowledge, the effect of chronic TMA administration on renal and cardiovascular systems has not been evaluated thus far. Therefore, the present study aimed to assess the impact of chronic TMA exposure on cardiovascular and renal systems in rats.

## Methods

### Animal

The study was performed according to Directive 1020/63 EU on the protection of animals used for scientific purposes and approved by the II Local Ethical Committee in Warsaw (permission: WAW2/098/2019). The study was performed on 12-week-old male Sprague–Dawley rats (SPRD). Rats were obtained from the Central Laboratory for Experimental Animals, Medical University of Warsaw, Poland.

Rats were housed in groups of 2–4 animals in propylene cages, fed a laboratory diet (Labofed B standard, Kcynia, Poland), 12 h light / 12 h dark cycle, temperature 22–23 °C and humidity 45–55%.

The animals were divided into 3 groups of 9 rats. The first group had access to tap water (control group), the second group to a TMA solution (Sigma-Aldrich, St. Louis, MO, USA) at a concentration of 4.85 mmol/L (TMA—low dose group—“L group”) and the third group to a TMA solution at a concentration of 14.24 mmol/L (TMA—high dose group—“H group”). Based on pilot experiments, the low dose of TMA was selected as a dose that did not increase urine TMA excretion (suggesting complete TMA oxidation to TMAO). The high dose of TMA was selected as a dose that increased TMA urine excretion and plasma concentration. The water or TMA solutions were available to animals ad libitum. The study ran for 18 weeks. Blinding of laboratory technicians were not possible due to characteristic smell of TMA in drinking water and body fluids [32].

### Metabolic, echocardiography and hemodynamic measurements

After 18 weeks, the animals were placed in metabolic cages for 48 h. The weight of water consumed, food consumed, feces excreted, urine excreted, and body weight were measured after 24 h and 48 h. 24 h urine collection was performed to measure water-electrolyte, TMA and TMAO balance and concentrations of choline, carnitine, TMA and TMAO. Fresh urine samples produced during spontaneous voids were collected to measure urine protein/creatinine and KIM-1 levels. The next day, an ECHO was performed (Samsung HM70: an ultrasound system equipped with a linear probe 5–13 MHz). After the ECHO study, the animals were anesthetized with urethane (1.5 g/kg BW). The femoral artery was cannulated for arterial blood pressure measurements with Biopac MP 150 (Biopac Systems, USA). After completing the measurements, blood was drawn from the heart to measure concentrations of serum choline, carnitine, TMA and TMAO, serum KIM-1 and other serum biochemical analysis. The rats were euthanized by cervical vertebrae dislocation. Colon feces were collected and prepared as previously described [33]. The heart, lung, kidney (separately cortex and medulla) and liver were collected and frozen at −80 °Celsius. The harvested fragments of the liver, kidneys and heart were fixed in a buffered solution of 10% formalin. The TMA, TMAO, choline and carnitine concentrations in stool, serum and urine were examined. Serum sodium, potassium, creatinine, urea and urine creatinine,protein and glucose were analyzed using a Cobas 6000 analyzer (Roche Diagnostics, Indianapolis, IN, USA).

### Stools, plasma, urine and tissue choline, carnitine, TMA and TMAO measurements

Tissue samples were weighed, placed in 10% ethanol (90 µL per 10 mg tissue) and homogenized using the Precellys Cryolys Evolution tissue homogenizer (Bertin Instruments). After homogenization, samples were stored at −80 °C until analysis.

Samples were prepared using the derivatization technique. The derivatization reaction of TMA was based on a modified Johnson’s protocol. The reader is referred to the Additional file 1: Methods for a detailed description of the protocol. Metabolite concentrations in serum, urine, stool extract and tissue homogenate were evaluated using Waters Acquity Ultra Performance Liquid Chromatograph coupled with Waters TQ-S triple-quadrupole mass spectrometer. The mass spectrometer was operated in multiple-reaction monitoring (MRM)-positive electrospray ionization (ESI +) mode for all analytes. Analyte concentrations (choline, carnitine, TMA and TMAO) were calculated using a calibration curve prepared by spiking water with working stock solutions. Biological samples (serum, urine, stool extract and tissue homogenate) were compared against the calibration curve. The concentrations of analytes (choline, carnitine, TMA and TMAO) in tissue were measured in dry tissue mass. For a detailed description of the method, see Additional file 1: Methods.

### Histopathology

The preserved tissues (kidney, liver and heart) were macroscopically examined and then dehydrated in graded ethanol and xylene baths. The dehydrated sections (measuring 3–4 µm) were then embedded in paraffin wax and stained with hematoxylin and eosin (H-E). The liver, heart and kidney tissue structures were examined using an Axiolab A5 light microscope with Axiocam 208 color and ZEN 3.0 software (Zeiss, Jena, Germany). Microscopic evaluation was performed at 10× and 40× magnification. Morphometric measurements of 5 arcuate arteries and 5 arterioles were performed for each individual. Four measurements were made at the ×40 lens magnification using the ZEN 3.0 software (Zeiss, Jena, Germany) for each type of vessel.

### ELISA test

Serum and urine KIM-1 (cat. No ab223858) levels were evaluated using EIAab Kits (Wuhan EIAab Science Co. Ltd., Wuhan, Hubei, China). Both protocols were performed according to the standard protocol by ELISA Kit Operating Instruction. The absorbance intensity was measured at 450 nm with a Multiskan microplate reader (Thermo Fisher Scientific, Waltham, MA, USA). All experiments were performed in duplicate.

### RNA isolation and RT-qPCR

Total cellular RNA was extracted from the lungs, liver, renal cortex and renal medulla (approximately 15 mg of wet tissue) using a Trizol reagent (Invitrogen, Carlsbad, CA, USA) according to Chomczyński and Sacchi [34]. The procedure was performed as previously described [35]. Specific primers were purchased from Bio-rad (Additional file 2: Table S1). The PCR products were subjected to a melting curve analysis to confirm amplification specificity. Bio-Rad CFX Maestro Software (Hercules, CA, USA) was used for data analysis. Transcript levels were normalized relative to the *Gapdh* reference gene (selected from four different housekeeping genes using NormFinder software: version 0.953, MOMA, Aarhus, Denmark) for each tissue separately.

### Statistic

The Shapiro–Wilk test was used to test the normality of the distribution. Differences between the three groups for metabolic, hemodynamic, and ECHO parameters and serum and urine KIM-1 concentrations, morphometric measurements of arcuate arteries and arterioles were evaluated by one-way ANOVA followed by Tukey’s post hoc test. Differences between the groups for urine protein, creatinine and glucose concentrations, metabolites concentrations (choline, carnitine, TMA, TMAO), TMA/TMAO balance, RT-qPCR analysis of FMO1, FMO3, FMO5, REN, AGT, AGTr1a, AGTr1b, AGT2 were evaluated by Kruskal–Wallis test followed by post-hoc Dunn’s test. A value of two-sided p < 0.05 was considered significant. Statistical analysis was conducted using STATISTICA 13.3 (Stat Soft, Krakow, Poland).

## Results

### Metabolic parameters

After the treatment, there were no significant differences between the groups in body weight, food intake and urine output. Rats on TMA tended to drink more water and excrete more feces (Table 1).

**Table 1:** Metabolic parameters

Parameter	Control group	L group	H group	One-way ANOVA
Body weight [g]	431.52 (± 20.08)	429.77 (± 25.56)	440.93 (± 21.22)	*P* = 0.53
Water [g/24 h]	31.94 (± 6.01)	36.28 (± 5.07)	35.47 (± 5.04)	*P* = 0.21
Food [g/24 h]	19.28 (± 1.85)	20.23 (± 1.79)	20.33 (± 2.70)	*P* = 0.53
Faces [g/24 h]	7.74 (± 2.33)	10.08 (± 2.38)	9.48 (± 1.66)	*P* = 0.08
Urine [g/24 h]	19.17 (± 5.64)	19.32 (± 3.53)	18.83 (± 3.75)	*P* = 0.97

All data are expressed as the mean ± SD. ANOVA followed by post hoc Tuckey-test

*L group* TMA low-dose group; *H group* TMA high-dose group.

### Hemodynamic and ECHO parameters

Rats on TMA showed higher blood pressure (Table 2). Specifically, the H group had significantly higher systolic blood pressure and demonstrated a trend towards higher diastolic blood pressure.

**Table 2:** Hemodynamic parameters

Parameter	Control group	L group	H group	One-way ANOVA
SBP [mmHg]	126.3 (± 11.4)	141.3 (± 17.9)	151.2 (± 19.9)*	*P* = 0.02
DBP [mmHg]	70.8 (± 14.0)	64.2 (± 13.5)	81.78 (± 14.1)	*P* = 0.06
Heart rate [bpm]	314 (± 86)	330 (± 58)	373 (± 13)	*P* = 0.16
SVR [mmHg⋅min⋅mL-1]	1.51 (± 0.95)	0.73 (± 0.19)	3.67 (± 5.16)	*P* = *0.14*

All data are expressed as the mean ±SD; ANOVA followed by post hoc Tuckey-test; *P < 0.05 vs control group

*SBP* systolic blood pressure, *DBP* diastolic blood pressure, *SVR* Systemic Vascular Resistance, *L group* TMA low-dose group, *H* *group* TMA high-dose group

There were no significant differences between the groups in echocardiographic parameters (Additional file 3: Table S2).

### TMA and TMAO concentrations in stool, serum and urine

The median TMA stool concentration was significantly higher in the H group than in the control or L group. The median TMA and TMAO serum concentrations were significantly higher in the H group than in the control group, eightfold and sixfold, respectively. TMA and TMAO serum concentrations were also higher in the L group than in the control group. The median TMA and TMAO urine concentrations were significantly higher in the H group than in controls, 17-fold and 24-fold, respectively. The median TMAO urine concentration was higher in the L group than in the control group [11-fold], but TMA urine concentration was similar to the control group (Table 3).

**Table 3:** TMAO, TMA and their precursors concentrations in stool, serum and urine

Parameter [µM/l]	Control group	L group	H group	Kruskal–Wallis test
Stool				
TMA	19.70 (13.86; 30.38)	30.14 (21.27; 41.67)	54.62 (47.88; 79.21)**	*P* = 0.003
Choline	77.55 (67.81; 92.10)	61.87 (57.19; 84.98)	81.52 (77.15; 88.23)	*P* = 0.41
Carnitine	8.91 (7.43; 11.49)	6.07 (3.97; 8.03)	6.52 (5.87; 7.62)	*P* = 0.26
Serum				
TMAO	8.96 (6.46; 11.85)	26.25 (25.30; 31.67)*	49.50 (45.84; 84.76)***	*P* < 0.001
TMA	0.04 (0.03; 0.04)	0.14 (0.09; 0.17)*	0.32 (0.27; 0.64)***	*P* < 0.001
Choline	75.92 (60.91; 104.79)	117.31 (93.88; 135.97)	95.77 (78.62; 110.24)	*P* = 0.13
Carnitine	66.77 (60.34; 70.64)	74.90 (64.88; 80.64)	66.47 (57.60; 73.78)	*P* = 0.29
Urine				
TMAO	584.98 (448.50; 686.60)	6 143.59 (5 569.76; 6 449.26)*	14 049.76 (13 902.77; 15 586.82)***; #	*P* < 0.001
TMA	15.18 (9.75; 20.99)	15.10 (12.94; 16.37)	257.37 (207.48; 324.83)***; ###	*P* < 0.001
Choline	94.41 (69.78; 151.54)	135.07 (118.75; 160.19)	137.25 (129.10; 154.52)	*P* = 0.27
Carnitine	169.45 (142.06; 232.12)	153.41 (141.67; 171.76)	134.18 (123.83; 165.68)	*P* = 0.23

All data are expressed as the median (Q1; Q3); Kruskal–Wallis test followed by post-hoc Dunn’s test

*TMA* trimethylamine,*TMAO* trimethylamine oxide, *L group* TMA low-dose group, *H group* TMA high-dose group

*P < 0.05 vs control group; ***P < 0.001 vs control group; ^#^P < 0.05 vs low dose group; ^###^P < 0.001 vs low dose group

There were no significant differences between groups in choline and carnitine concentrations. TMAO concentration in stool was below the lower limit of quantification.

### TMA/TMAO balance

**Fig. 1:**
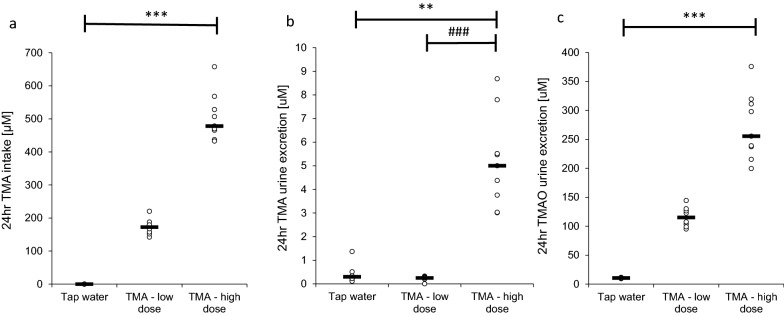
TMA/TMAO balance. **a** the amount of TMA consumed per day; **b** the amount of TMA excreted per day; **c** the amount of TMAO excreted per day in rats maintained either on tap water (control group) or low and high dose of TMA. TMA, trimethylamine; TMAO, trimethylamine oxide. All data are expressed as the median (n = 9); Kruskal–Wallis test followed by post-hoc Dunn’s test; ***P < 0.001 vs tap water group; ^###^P < 0.001 vs low dose group

L and H groups consumed TMA in water at an average dose of 200 and 500 µM/day, respectively. The amount of TMA excreted per day was significantly higher in the H group than in the control or L group, 17-fold and 20-fold, respectively. The amount of TMAO excreted per day was significantly higher in H and L groups than in the control group (Fig. 1), 24-fold and 11-fold, respectively.

### TMA and TMAO concentrations in the tissue

In most of the evaluated tissues, TMAO concentrations were several-fold higher in TMA groups than in controls. In contrast, TMA levels were only moderately higher in TMA groups and there were no significant differences in choline and carnitine levels. (Table 4).

**Table 4:** The TMAO, TMA and other metabolites concentrations in the tissue

Parameter [µM/kg]	Control group	L group	H group	Kruskal–Wallis test
Lungs				
TMAO	9.27 (5.19; 11.07)	52.04 (48.16; 57.69)*	218.25 (191.74; 282.54)***	*P* < 0.001
TMA	6.52 (5.14; 8.93)	6.71 (5.85; 8.63)	9.76 (7.26; 11.28)	*P* = 0.18
Choline	1 870.59 (1 784.86; 2 062.80)	2 038.94 (1 985.86; 2 159.95)	1 969.03 (1 877.74; 2 120.19)	*P* = 0.42
Carnitine	307.11 (277.93; 309.62)	279.62 (272.50; 342.72)	309.68 (293.66; 334.90)	*P* = 0.23
Liver				
TMAO	5.40 (4.24; 9.57)	19.82 (13.79; 23.19)	35.85 (29.32; 41.87)***	*P* = 0.001
TMA	42.80 (38.09; 47.25)	63.12 (53.61; 73.26)*	80.39 (62.98; 92.61)***	*P* < 0.001
Choline	1 112.92 (921.26; 1 462.16)	1 124.02 (814.50; 1 241.82)	1 265.63 (1 014.44; 2 007.82)	*P* = 0.11
Carnitine	470.73 (452.88; 481.55)	514.03 (477.57; 589.24)	568.35 (498.70; 589.18)	*P* = 0.34
Renal cortex				
TMAO	14.48 (13.13; 21.87)	27.76 (26.04; 36.82)	189.74 (82.22; 204.48)***	*P* < 0.001
TMA	158.48 (149.59; 163.61)	240.14 (229.81; 262.72)*	487.66 (389.55; 593.86)***	*P* < 0.001
Choline	1 441.73 (1 159.19; 1 538.25)	1 512.66 (1 462.91; 1 687.80)	1 346.40 (1 025.19; 1 521.07)	*P* = 0.56
Carnitine	314.85 (291.49; 362.62)	344.41 (226.85; 364.37)	358.71 (306.06; 434.33)	*P* = 0.78
Renal medulla				
TMAO	18.53 (14.43; 27.82)	42.74 (34.16; 43.01)	192.02 (73.95; 213.70)***, #	*P* < 0.001
TMA	193.08 (186.32; 218.50)	286.22 (267.48; 294.91)	449.83 (348.32; 524.00)***	*P* < 0.001
Choline	2 408.67 (1 737.76; 2 570.56)	1 198.82 (399.11; 1 493.18)	1 751.37 (1 308.89; 2 353.36)	*P* = 0.06
Carnitine	341.55 (314.27; 355.18)	297.82 (288.73; 310.64)	306.90 (299.75; 355.56)	*P* = 0.10
Heart				
TMAO	3.22 (2.45; 5.77)	35.59 (34.37; 48.97)*	96.30 (90.30; 112.43)***	*P* < 0.001
TMA	0.70 (0.58; 0.85)	4.02 (3.67; 4.36)*	11.32 (10.42; 13.17)***, #	*P* < 0.001
Choline	129.98 (124.15; 156.68)	145.91 (134.83; 150.98)	140.36 (135.37; 152.92)	*P* = 0.45
Carnitine	759.22 (727.41; 796.04)	795.12 (698.43; 838.37)	883.12 (814.61; 916.01)	*P* = 0.06

*TMA*, trimethylamine; *TMAO*, trimethylamine oxide; L group – TMA low-dose group; H group – TMA high-dose group. All data are expressed as the median (Q1; Q3); Kruskal–Wallis test followed by post-hoc Dunn’s test. *P < 0.05 vs control group; ***P < 0.001 vs control group; #P < 0.05 vs low dose group

### Urine biochemical analysis

**Fig. 2:**
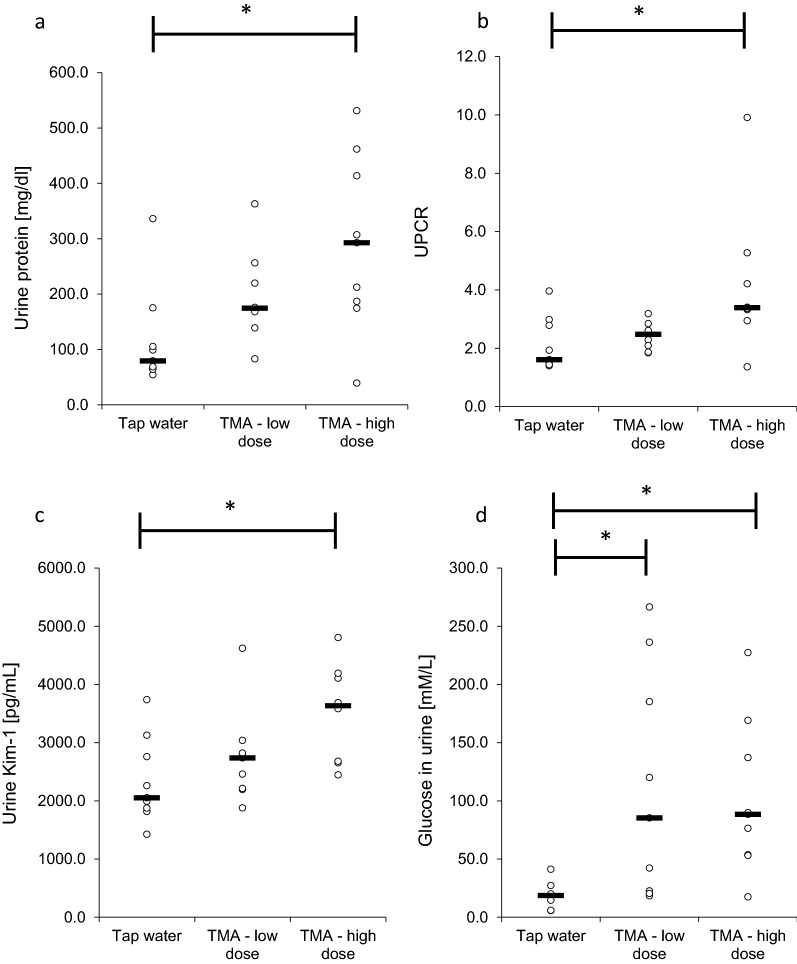
Urine biochemical analysis. **a** protein concentration in urine; **b** protein to creatinine ratio in urine; **c** Kim-1 concentration in urine; **d** glucose concentration in urine in rats maintained either on tap water (control group) or low and high dose of TMA. *TMA* trimethylamine, *UPCR* urine protein creatinine ratio. All data are expressed as the median (n = 9); Kruskal–Wallis test followed by post-hoc Dunn’s test for urine protein, UPCR and urine glucose; ANOVA followed by post-hoc Tuckey test for urine Kim-1; *P < 0.05 vs tap water group

Rats from the H group showed significantly higher protein concentration in urine, protein/ creatinine ratio and glucose concentration in urine than the control group (Fig. 2). Urine KIM-1 levels were significantly higher in the H group compared to the control group.

### Serum biochemical analysis

There was a trend for a higher serum potassium concentration in the TMA groups than in the control group (Additional file 4: Table S3). There were no significant differences between groups in the serum urea, serum creatinine, serum sodium concentrations, creatinine clearance and KIM-1 protein in serum.

### FMOs genes expression

**Fig. 3:**
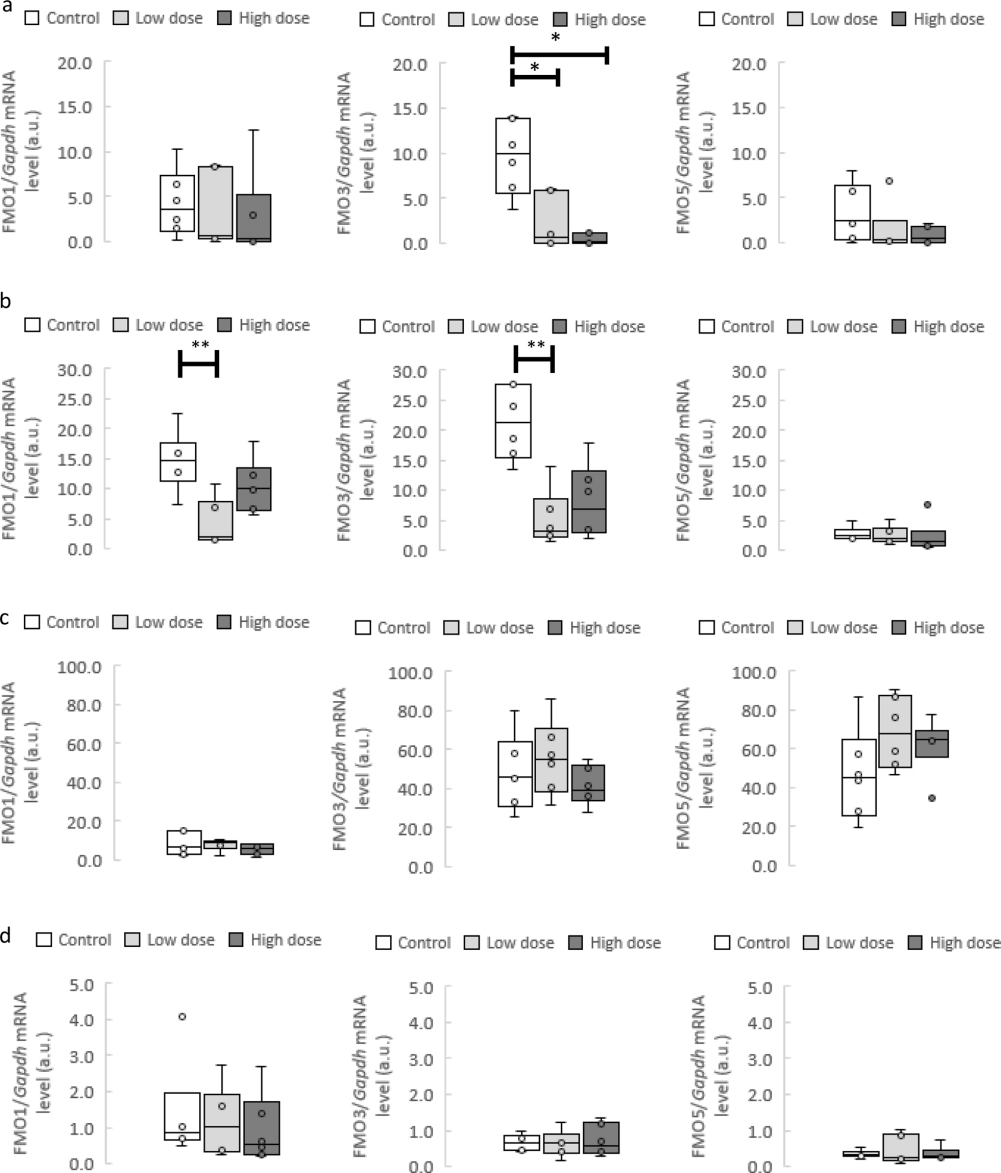
FMOs genes expression. FMOs in the kidney, liver and lungs. RT-qPCR analysis of FMO1, FMO3 and FMO5 transcript levels in the **a** renal cortex, **b** renal medulla, **c** liver and **d** lungs in rats maintained either on tap water (control group) or low and high dose of TMA. *TMA* trimethylamine; *FMO* Flavin-containing monooxygenase. All data are expressed as the median, Q1, Q3, MIN, MAX (n = 6; use arbitrary units); Kruskal–Wallis test followed by post-hoc Dunn’s test. *P < 0.05 vs tap water group; **P < 0.01 vs tap water group

The FMO3 gene expression in the renal cortex and renal medulla was significantly lower in the L group than in the control group. The L group had significantly lower FMO1 gene expression in the renal medulla than the control group. Additionally, the H group had significantly lower FMO3 gene expression in the renal cortex than the control group. There were no significant differences between groups in the FMO5 gene expression in evaluated tissues (Fig. 3).

### Renin-angiotensin system gene expression

The AGT gene expression in the renal medulla was significantly lower in the L group than in the control group. The AGTr1b gene expression in the renal cortex was increased in the L group compared to the control group. There were no significant differences between groups in the REN, AGTr1a, and AGTr2 gene expression (Additional file 5: Fig. S1).

### Histopathological evaluation

The control group showed no pathological changes in the liver or heart tissue. Nevertheless, in the kidneys of control animals, we observed some lymphohistiocytic infiltrates in the stroma, Bowman's capsule hyperplasia and thickening of the basal membranes of the tubules and vessels of the glomerulus.

**Fig. 4:**
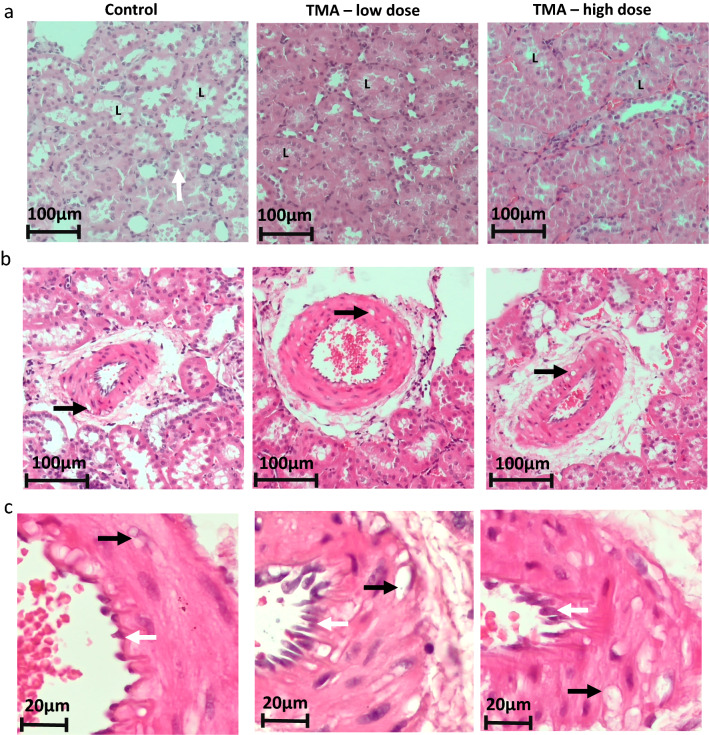
Histopathology of the kidney. **a** Renal convoluted tubules (original magnification 10X, H&E stain), **b** renal medium size artery (original magnification 10×, H&E stain), **c** renal medium size artery (original magnification 40×, H&E stain) in rats maintained either on tap water (control group) or low and high dose of TMA. *TMA* trimethylamine, *L* lumen of convoluted renal tubules, Black arrow – vacuoles of various sizes within the cell cytoplasm, White arrow – endothelium

In the kidneys of the L and H groups, the severity of the abovementioned changes was greater. Specifically, in the L and H groups, the pronounced thickening of the membranes of the substate tubules and glomerular vessels, mild degeneration of renal bodies with glomerulosclerosis, the hypertrophy of the tunica media of arteries and arterioles with vacuolization and degeneration of smooth myocytes were present. The H group showed significantly thicker accurate arteries and arterioles (45.05 ± 2.90 µm and 23.79 ± 2.60 µm; respectively) than the control group (36.61 ± 0.15 µm, P = 0.001 and 18.44 ± 0.62 µm, P = 0.006; respectively) and the L group (40.47 ± 1.77 µm, P = 0.02 and 20.16 ± 0.99 µm, P = 0.02; respectively; Additional file 6: Fig. S2). Moreover, mild to moderate chronic progressive nephropathy (CPN) can be described in rats from L and H groups. These changes are more pronounced in the H group (Fig. 4).

**Fig. 5:**
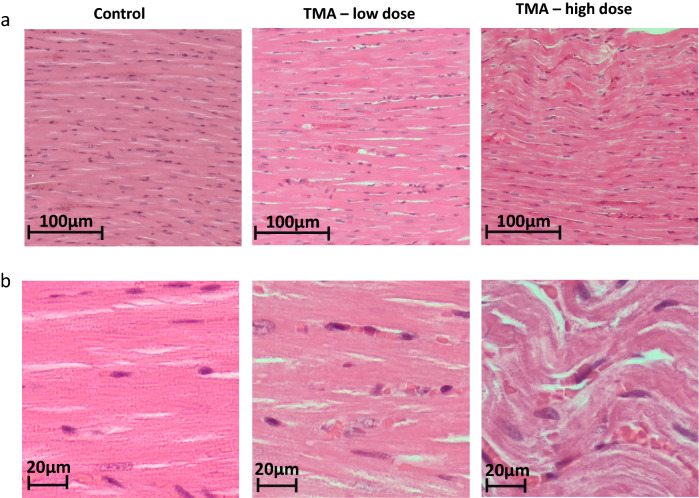
Histopathology of the heart. **a** Myocardium of left ventricle (original magnification 10×, H&E stain), **b** myocardium of left ventricle (original magnification 40X, H&E stain) in rats maintained either on tap water (control group) or low and high dose of TMA. *TMA* trimethylamine

No pathological changes were found in the liver of the L group. Rats from the H group showed slight to moderate hepatocyte edema and cytoplasmic vacuolization, indicative of hydropic degeneration with features of acid degeneration. Moreover, there is marginalization of chromatin in the hepatocytes’ nuclei as well as prominent large 1–3 nucleoli. In the control and L groups, no significant lesions in the heart were found. In the H group in the myocardium, a focal wave course of cardiomyocytes, reduced visibility of cytoplasm striation and slight vacuolation of the cytoplasm were observed (Fig. 5).

## Discussion

The novel finding of our study is that chronic administration of TMA causes proteinuria, elevated urine KIM-1 and glucose levels, histological characteristics of chronic progressive nephropathy and increased blood pressure in rats. These results suggest a deleterious effect of TMA on the kidneys and cardiovascular system.

Recently, the effects of TMAO on the cardiovascular and renal systems have been researched extensively [36, 37]. Some studies suggest that TMAO may contribute to the development of cardiorenal syndrome [3, 4]. On the other hand, it has been well established that TMAO is one of the osmolytes protecting proteins from high osmotic pressure [38, 39]. For instance, TMAO and other osmolytes such as betaine, sorbitol and glycerophosphorylcholine play a protective role in the renal medulla in which osmolality exceeds plasma osmolality by up to 4–5-folds [40–42].

In our study, chronic administration of TMA caused proteinuria, glucosuria and elevated KIM-1 levels in urine (Fig. 2). Proteinuria is a well-known marker of kidney damage [43, 44], and is associated with CVD [45, 46]. KIM-1 is a transmembrane tubular protein, which is up-regulated and present in urine after the renal tubular injury, both in rats and humans [47, 48]. This protein is also a marker for drug-induced kidney toxicity [49]. Glucosuria is a common finding in diabetes mellitus and diabetic kidney disease but it may also be present in nondiabetic advanced CKD [50]. In contrast to humans, in rats glucose is observed also in healthy animals [51–53]. Notably, in this study the urine glucose concentration was significantly higher in the TMA groups than in the control group, which further points to tubular damage in rats exposed to TMA in drinking water. The chronic administration of TMA also caused histopathological changes, including thickening of the basement membranes and increased infiltration of mononuclear cells without signs of concomitant inflammation, which may reflect CPN (Fig. 4). CPN is a spontaneous disease with unknown etiology. The observed changes in the renal parenchyma in rats receiving TMA may result from either the activation or aggravation of the ongoing CPN due to a direct effect of TMA on the kidneys or may be secondary to TMA-induced hemodynamic disturbances [54–56].

The association between CKD and hypertension is widely studied and hypertension may be a cause and a consequence of kidney disease [57–59]. Herein we observed that rats administered TMA showed increased arterial blood pressure (Table 2). Specifically, a significant increase in systolic blood pressure was accompanied by a more moderate increase in diastolic blood pressure. Systemic vascular resistance calculated from cardiac output and mean arterial blood pressure tended to be higher in rats exposed to the higher dose of TMA, which suggest that in this group the increase in BP was in part mediated by vasoconstriction. In general, these findings align with our previous studies, showing hypertensive response after the acute administration of TMA [22]. In this study, we did not find indications of fluid retention or water-electrolyte disturbances. However, rats on TMA tended to drink more water and show elevated serum potassium levels. Renal regulation of water-electrolyte balance and blood pressure is exerted mainly through the renin-angiotensin system. However, we did not find any consistent changes in the RAS gene expression in the kidneys that could explain the effect of TMA on blood pressure and water-electrolyte balance (Additional file 5: Fig. S1). Finally, we did not find any significant changes in echocardiographic parameters (Additional file 3: Table S2). Specifically, TMA and control groups did not differ in the atria and ventricle size, stroke volume or ejection fraction. Therefore, it seems that the magnitude or/and duration of hemodynamic changes were insufficient to produce noticeable remodeling of the heart.

There are some clinical data linking high TMA levels to renal system pathologies. Hsu et al. showed that children with end-stage renal failure had higher plasma concentration of TMA than patients with stage 1 chronic kidney disease. They also demonstrated that plasma TMA levels were inversely correlated with high BP and eGFR [8]. Research also shows that patients with end-stage renal disease have increased plasma TMA concentration [11] and TMA levels in exhaled air [28], but these levels decrease after dialysis. There is also evidence that patients with trimethylaminuria due to FMO3 deficiency show higher blood pressure [60].

However, the effects of TMA on the renal and cardiovascular systems have not been studied so far in interventional studies. To the best of our knowledge, our study is the first showing the negative effect of TMA on the kidneys; a finding which suggests a causative relationship between TMA accumulation due to kidney failure and cardiorenal complications of CKD. It may be speculated that TMA is one of the mediators in the vicious cycle of the cardiorenal syndrome.

Although there are multiple studies evaluating TMAO and TMA concentrations in fish and other marine animals’ muscle and liver tissues [39, 61–64], studies showing TMAO and TMA concentration in tissue of mammals are scant or lacking. Present work broadens the knowledge of TMA and TMAO tissue distribution in mammals. In the current study, control animals showed the highest TMA and TMAO concentrations in the renal cortex (158.5 µM/kg and 14.5 µM/kg, respectively) and renal medulla (193.1 µM/kg and 18.5 µM/kg, respectively, Table 4). The lowest TMA and TMAO concentrations were found in the heart (0.7 µM/kg and 3.22 µM/kg, respectively). The liver contained higher levels of TMA (42.8 µM/kg) than TMAO (5.4 µM/kg). TMA concentration (6.5 µM/kg) in the lungs was lower than TMAO concentration (9.3 µM/kg).

Three decades ago, Da Costa et al. assessed TMA and TMAO concentrations in the rat’s liver and kidney [65] but using different methods. They measured concentrations in tissue wet mass, not in tissue dry mass as in our study, making the comparison of Da Costas and our results difficult. TMA concentration was higher in the liver (437 nM/g) and kidney in the Da Costa study (531 nM/g) than in our study. TMAO concentration in the liver was over 100-fold higher (633 nM/g) than in our study, while TMAO concentration in the kidneys was not provided by Da Costa et al. It needs to be stressed that, in contrast to relatively simple and reproducible measurements of TMAO levels across numerous studies [4, 8, 10, 14, 16, 18–21, 66], the measurements of TMA are much more difficult. There are discrepancies in reported absolute TMA levels across the few available studies [11, 23, 24, 33, 35, 65, 67–69].

It needs to be noted, that the determination of small volatile amines such as TMA or ammonium is very challenging [67]. In this study, we used the method based on the derivatization technique which gives different TMA plasma results than the methods used before [21, 33]. We suspect that the method without derivatization gives higher plasma levels of TMA due to the presence of analytes containing TMA in their structure which coelute with trimethylamine and break down at the ion source yielding falsely TMA results. After several years of experience with TMA and TMAO determination in various tissues, we believe that the derivatization method is more appropriate because it allows reducing the volatility of trimethylamine and transforms trimethylamine into more stable and more compatible to liquid chromatography derivative before injection. For a detailed description of the method, see Additional file 1: Methods.

After crossing the gut-blood barrier, TMA is metabolized in the liver to TMAO by FMOs, mainly FMO3 [27, 70]. Five isoforms of FMOs are expressed in humans and animals. FMO3 shows the highest expression in the human liver whereas the human kidneys show the highest expression of FMO1 [27]. Rats also show FMO1 and FMO3 expression in the liver and kidneys [71–73]. Novick et al. observed higher FMO1 and FMO3 expression in the kidneys than in the liver. FMO1 expression was highest in the proximal and distal tubules, whereas FMO3 expression was highest in the distal tubules, collecting tubules and glomeruli [73].

To the best of our knowledge, our study is the first to evaluate the expression of FMO1, FMO3 and FMO5 after chronic TMA administration (Fig. 3). Our study shows that chronic TMA administration reduces FMO3 expression in the renal cortex and FMO1 and FMO3 expression in the renal medulla. Due to the accumulation of products, the negative feedback mechanism is often found in the regulation of enzyme, hormone and receptor activity in human and animal organisms [74–78]. Therefore, we speculate that rapid oxidation of TMA to TMAO caused downregulation of FMO1 and FMO3 expression in the kidney. Future research on TMA to TMAO oxidation in the kidneys is needed.

Finally, our study, suggests that TMA is rapidly oxidized to TMAO, which may limit the toxic effect of TMA on other organs. Namely, serum TMA concentrations in all groups were at least 100 times lower than TMAO concentrations. Furthermore, administration of TMA resulted in small increases in TMA tissue levels but 7–30-fold increases in TMAO levels (depending on the type of tissue, for comparison of the control group and the H group). Moreover, 24-h urine excretion of TMAO was 11 and 24 times higher in TMA-treated rats (the L group and the H group, respectively). In contrast, TMA 24-h urine excretion increased 17 times only for the H group (in the L group, we did not notice any changes).

## Conclusion

In conclusion, chronic TMA administration at high doses increases blood pressure and causes deleterious effects on the kidneys, including proteinuria, increased urine KIM-1 and glucose levels in rats. TMA is rapidly oxidized to TMAO, which may limit the toxic effect of the molecule. Furthermore, TMA lowers the expression of FMO1 and FMO3 in the kidneys. More research on TMA, in particular in pathological states characterized by impaired TMA to TMAO oxidation, is needed to understand the role of TMA and TMAO in the pathological process of cardiorenal syndrome.

## Supplementary Information

**Additional file 1. ** Methods

**Additional file 2: Table S1.** List of oligonucleotide primers used for RT-qPCR

**Additional file 3: Table S2.** Echocardiographic parameters. IVSD, interventricular septum thickness at diastole; LVDD, left ventricular diastolic diameter; PWD, posterior wall thickness at diastole; IVSS, interventricular septum thickness at systole; LVDS, left ventricular systolic diameter; PWS, posterior wall thickness at systole; EF, ejection fraction; FS, fractional shortening, LVEDV, left ventricular end-diastolic volume; LVESV, left ventricular end-systolic volume; AO, aortic root diameter; LA, left atrium diameter; CO; cardiac output; L group – TMA low-dose group; H group – TMA high-dose group. All data are expressed as the mean ± SD; ANOVA followed by post hoc Tuckey-test

**Additional file 4: Table S3.** Serum biochemical analysis. L group – TMA low-dose group; H group – TMA high-dose group. All data are expressed as the mean ± SD

**Additional file 5: Fig. S1.** Renin – angiotensin system. The genes of the renin-angiotensin system. RT-qPCR analysis of REN, AGT, AGTr1a, AGTr1b, AGTr2 transcript levels in the (A) renal cortex, (B) renal medulla, (C) liver in rats maintained either on tap water (control group) or low and high dose of TMA. Abbreviations: TMA, trimethylamine; REN, renin; AGT, angiotensinogen; AGTr1a, angiotensin II receptor, type 1a; AGTr1b, angiotensin II receptor, type 1b; AGTr2, angiotensin II receptor, type 2. All data are expressed as the median, Q1, Q3, MIN, MAX (n = 6; use arbitrary units); Kruskal–Wallis test followed by post-hoc Dunn’s test. **P < 0.01 vs control group

**Additional file 6: Fig. S2.** Morphometry. Morphometric measurement of renal (a) arteries and (b) arterioles in rats maintained either on tap water (control group) or low and high dose of TMA. Abbreviations: TMA, trimethylamine. Morphometric measurements of five arcuate arteries and five arterioles were performed for each individual. Four measurements were made for each vessel. All measurements are expressed as single points (n = 3–5); ANOVA followed by post-hoc Tuckey test; *P < 0.05

## Data Availability

The part of the raw data generated or analyzed during this study are included in this published article and its supplementary information files. The raw data presented in tables are available from the corresponding author upon on reasonable request.

## Abbreviations

AGT: Angiotensinogen
AGTr: Angiotensinogen receptor
AO: Aortic root diameter
CKD: Chronic kidney disease
CO: Cardiac output
CPN: Chronic progressive nephropathy
CRS: Cardiorenal syndrome
DBP: Diastolic blood pressure
EF: Ejection fraction
FMO: Flavin-containing monooxygenase
FS: Fractional shortening
IVSD: Interventricular septum thickness at diastole
IVSS: Interventricular septum thickness at systole
KIM-1: Kidney injury molecule 1
LA: Left atrium diameter
LVDD: Left ventricular diastolic diameter
LVDS: Left ventricular systolic diameter
LVEDV: Left ventricular end-diastolic volume
LVESV: Left ventricular end-systolic volume
PWD: Posterior wall thickness at diastole
PWS: Posterior wall thickness at systole
RAS: Renin angiotensin system
REN: Renin
SBP: Systolic blood pressure
SPRD: Sprague–Dawley rats
SV: Stroke volume
SVR: Systemic vascular resistance
TMA: Trimethylamine
TMAO: Trimethylamine oxide
UPCR: Urine protein creatinine ratio
