# Supplementary material for: Trimethylamine, a gut bacteria metabolite and air pollutant, increases blood pressure and markers of kidney damage including proteinuria and KIM-1 in rats
Source: J Transl Med. 2022 Oct 15;20:470. doi: 10.1186/s12967-022-03687-y (PMC9571686; doi:10.1186/s12967-022-03687-y)
Supplement: Supplementary file 1 — Additional file 1. Methods [file 12967_2022_3687_MOESM1_ESM.doc]

**Additional methods**

**Chemicals**

Trimethylamine hydrochloride (TMA), trimethylamine-^13^C_3_,^15^N hydrochloride, trimethylamine N-oxide dihydrate (TMAO), L-carnitine hydrochloride and L-carnitine-(methyl-d_3_) inner salt, choline chloride, *tert*-butyl bromoacetate (TBBA), ammonia solution were purchased from Sigma Aldrich (Sigma-Aldrich, St. Louis, MO, USA). Trimethylamine N-oxide-D_9_ was obtained from Cambridge Isotope Laboratories, Inc (Cambridge Isotope Laboratories, Inc, Tewksbury, Massachusetts, USA). All stock solutions were prepared in methanol freshly. LC-MS grade acetonitrile, HPLC grade acetone, HPLC grade acetonitrile, HPLC grade methanol and formic acid were obtained from J.T. Baker (J.T. Baker, Phillipsburg, New Jersey, USA). Ultra-pure water (Mili-Q water) was produced by a water purification system (Mili-Q, Millipore, Milford, MA, USA).

**Sample preparation**

Tissue samples were weighed, placed in 10% ethanol (90 µL per 10 mg tissue) and homogenized using Precellys Cryolys Evolution tissue homogenizer (Bertin Instruments). After homogenization samples were stored in -80⁰C until analysis.

Samples were prepared using the derivatization technique. The derivatization reaction of TMA was based on Johnson’s protocol with modification(1). As a derivative agent *tert*-butyl bromoacetate was used. To 20 µL of a sample (biological and calibration samples), 10 µL of 2.5% ammonia solution and 50 µl of acetone (containing internal standards) were added and mixed. To create a trimethylamine derivative 50 µL of TBBA in acetonitrile was added and the mixture was incubated at room temperature (structure of derivative Fig. 1). The derivatization reaction was stopped after 30 min by the addition of 25 µl of 0.5% formic acid in 50% acetonitrile. Next, the solution was centrifuged and an aliquot was injected into the apparatus.


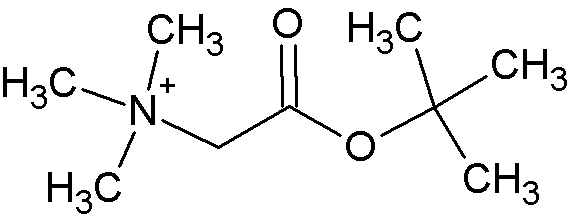


Fig. 1 Structure of TMA derivative

**Analyzes**

The instrumentation consisted of a Waters Acquity Ultra Performance Liquid Chromatograph (Waters, Milford, Massachusetts, USA) coupled with Waters TQ-S triple-quadrupole mass spectrometer (Waters, Manchester, UK). For the instrument control and data acquisition Waters MassLynx software was used (Waters, Manchester, UK). Waters TargetLynx was used to processed data (Waters, Manchester, UK).

Chromatographic separation was performed using a Waters HILIC column (1.7µm, 2.1mm x 50mm) (Waters, Milford, Massachusetts, USA) thermostatted at 60⁰C. Mobile phase A was 15mM ammonium formate in Mili-Q water, and mobile phase B was acetonitrile. The flow rate of the mobile phase was set at 0.5mL/min. The total time of separation was 2.2mins. The chromatographical gradient scheme is presented in Table 1.

Table 1. LC method gradient

| Time [min] | Flow [mL/min] | %A | %B |
| --- | --- | --- | --- |
| - | 0.5 | 10.0 | 90.0 |
| 1.3 | 0.5 | 30.0 | 70.0 |
| 1.5 | 0.5 | 10.0 | 90.0 |

The mass spectrometer was operated in multiple-reaction monitoring (MRM)- positive electrospray ionization (ESI+) mode for all analytes. For all analyzed compounds mass spectrometer optimized settings were as follows: capillary voltage = 2.5 kV, desolvation temperature = 350 ºC, desolvation gas flow = 550 L/h, cone gas flow = 150 L/h, nebuliser gas pressure = 7.0 bar, source temperature = 150 °C. MRM transitions, cone voltages, collision energies and retention times used in the described method are presented in Table 2. The first MRM transition of each compound served as a quantitative transition, the second as a confirmation transition.

Table 2. Monitored transitions for analytes, cone voltages, collision energies, retention times and LOQ of analyzed compounds

| Analyte | MRM transition | Cone voltage [kV] | Collision energy | Retention time [min] | LOQ  [ng/ml] |
| --- | --- | --- | --- | --- | --- |
| TMA derivative | 174.26>118.14 (qt)  174.26>59.06 | 20  20 | 15  20 | 0.92 | 0.5 |
| TMA IS derivative | 178.23>122.08  178.23>63.1 | 20  20 | 15  25 | 0.92 | - |
| TMAO | 76.076>57.97 (qt)  76.076>58.97 | 15  15 | 20  20 | 1.83 | 20 |
| TMAO-D9 (TMAO IS) | 85.13>68.2  85.13>66.2 | 20  20 | 10  14 | 1.83 | - |
| Choline | 104.08>60 (qt)  104.08>44.96 | 20  20 | 25  25 | 1.25 | 100 |
| L-carnitine | 162.11>85.06  162.11>103.04 qt | 20  20 | 20  15 | 1.74 | 5 |
| L-carnitine-(methyl-d_3_) (L-carnitine IS) | 165.13>85.02  165.13>103.16 qt | 20  20 | 20  15 | 1.74 | - |

The concentrations of analytes were calculated using calibration standard mix derived from a series of calibrator samples by spiking standard stock solutions into water. Calibration curves were generated by compared a ratio of the peak area of the analyzed compound to the peak of the internal standard against known analyte concentrations. Biological samples (plasma, urine, stool extract) were compared with an obtained calibration curve. The mean R2 coefficients of calibration curves were not lower than 0.98. Limits of quantification analytes are presented in Table 1.

**General Considarations**

Determination of small volatile amines such as trimethylamine (TMA) or ammonium is very difficult, not only using liquid chromatography. Gas chromatography seems to be the most appropriate method for TMA determination, but the authors also report some problems with this technique(2). There is currently no reliable method for the qualification of trimethylamine level. What’s more, most of the works where the concentration of TMA was determined concern urine samples, and relatively only a few studies determine the concentration of TMAO and TMA in plasma samples(3)^,(4)^. Usually, as an indicator of the level of trimethylamine in plasma and cardiovascular risks, trimethylamine oxide (TMAO) is measurement(5). TMAO is more compatible with LC-MS/MS because it is more stable and less volatile than TMA.

In this work, we used the method based on the derivatization technique which gives different TMA plasma results than the method used before. We suspect that the method without derivatization gives higher plasma levels of TMA due to the presence of analytes containing TMA in their structure which coelute with trimethylamine and break down at the ion source yielding falsely TMA results. Unfortunately, we are unable to identify these substances. Based on the latest publication and our experience with TMA and TMAO determination, it seems like the derivatization method is more appropriate because it allows reducing the volatility of trimethylamine and transforms trimethylamine into more stable and more compatible to liquid chromatography derivative before injection. Additionally currently, most of the works, where trimethylamine is determined using LC-MS/MS, based on derivatization technique.

Sample derivatization is a general term used for a chemical transformation designed to improve analytical capabilities. During derivatization, a chemical compound is converted into a product of a similar chemical structure: the derivative. Generally, a specific functional group of the compound participates in the derivatization reaction and transforms it into a derivate of deviating reactivity, solubility, boiling point, melting point, aggregate state, or chemical composition. New chemical properties can be used for separation and quantification. Derivatization in liquid chromatography allows to reduce the volatility of the tested substances, increase the mass, and improve the chromatographic parameters (selectivity, resolution, peak shape). The best derivatization agent quickly and completely reacts with the analyte and forms derivative which is stable under the chromatographic conditions. The reaction conditions should be mild and the analytical signal of the reagent should not interfere with the signal of the derivative. The derivatives and the reagent should be well soluble in the mobile phase.

References

1. Johnson DW. A flow injection electrospray ionization tandem mass spectrometric method for the simultaneous measurement of trimethylamine and trimethylamine N-oxide in urine. J Mass Spectrom. 2008;43(4):495-9.

2. Bain MA, Faull R, Fornasini G, Milne RW, Schumann R, Evans AM. Quantifying trimethylamine and trimethylamine-N-oxide in human plasma: interference from endogenous quaternary ammonium compounds. Anal Biochem. 2004;334(2):403-5.

3. Jia X, Osborn LJ, Wang Z. Simultaneous Measurement of Urinary Trimethylamine (TMA) and Trimethylamine N-Oxide (TMAO) by Liquid Chromatography-Mass Spectrometry. Molecules. 2020;25(8).

4. Veeravalli S, Karu K, Phillips IR, Shephard EA. A highly sensitive liquid chromatography electrospray ionization mass spectrometry method for quantification of TMA, TMAO and creatinine in mouse urine. MethodsX. 2017;4:310-9.

5. Dong Z, Liang Z, Guo M, Hu S, Shen Z, Hai X. The Association between Plasma Levels of Trimethylamine N-Oxide and the Risk of Coronary Heart Disease in Chinese Patients with or without Type 2 Diabetes Mellitus. Dis Markers. 2018;2018:1578320.
