# Supplementary material for: Trimethylamine, a gut bacteria metabolite and air pollutant, increases blood pressure and markers of kidney damage including proteinuria and KIM-1 in rats
Source: J Transl Med. 2022 Oct 15;20:470. doi: 10.1186/s12967-022-03687-y (PMC9571686; doi:10.1186/s12967-022-03687-y)
Supplement: Supplementary file 2 — Additional file 2: Table S1. List of oligonucleotide primers used for RT-qPCR [file 12967_2022_3687_MOESM2_ESM.docx]

**Additional Table1.** List of oligonucleotide primers used for RT-qPCR

| **Gene** | **Encoded product** | **Unique Biorad Assay ID** | **PCR product size [bp]** | **Ensembl Accession**  **number** |
| --- | --- | --- | --- | --- |
| *Fmo1* | flavin containing dimethylaniline monoxygenase 1 | qRnoCID0008990 | 103 | ENSRNOG00000034191 |
| *Fmo3* | flavin containing dimethylaniline monoxygenase 3 | qRnoCID0003196 | 92 | ENSRNOG00000003620 |
| *Fmo5* | flavin containing dimethylaniline monoxygenase 5 | qRnoCID0053250 | 114 | ENSRNOG00000018076 |
| *Ren* | Renin | qRnoCID0008721 | 115 | ENSRNOG00000002937 |
| *Agt* | Angiotensinogen | qRnoCED0051666 | 95 | ENSRNOG00000018445 |
| *Agtr1a* | Type-1A angiotensin II receptor | qRnoCID0052626 | 68 | ENSRNOG00000018346 |
| *Agtr1b* | Type-1B angiotensin II receptor | qRnoCED0005729 | 90 | ENSRNOG00000010640 |
| *Agtr2* | Type-2 angiotensin II receptor | qRnoCED0007551 | 83 | ENSRNOG00000050006 |
| *Gapdh* | glyceraldehyde-3-phosphate dehydrogenase | qRnoCID0057018 | 115 | ENSRNOG00000018630 |
