# Supplementary material for: Trimethylamine, a gut bacteria metabolite and air pollutant, increases blood pressure and markers of kidney damage including proteinuria and KIM-1 in rats
Source: J Transl Med. 2022 Oct 15;20:470. doi: 10.1186/s12967-022-03687-y (PMC9571686; doi:10.1186/s12967-022-03687-y)
Supplement: Supplementary file 3 — Additional file 3: Table S2. Echocardiographic parameters. IVSD, interventricular septum thickness at diastole; LVDD, left ventricular diastolic diameter; PWD, posterior wall thickness at diastole; IVSS, interventricular septum thickness at systole; LVDS, left ventricular systolic diameter; PWS, posterior wall thickness at systole; EF, ejection fraction; FS, fractional shortening, LVEDV, left ventricular end-diastolic volume; LVESV, left ventricular end-systolic volume; AO, aortic root diameter; LA, left atrium diameter; CO; cardiac output; L group – TMA low-dose group; H group – TMA high-dose group. All data are expressed as the mean ± SD; ANOVA followed by post hoc Tuckey-test [file 12967_2022_3687_MOESM3_ESM.docx]

Additional Table 2. – Echocardiographic parameters

| **Parameter** | **Control group** | **L group** | **H group** | **One-way ANOVA** |
| --- | --- | --- | --- | --- |
| IVSD [cm] | 0.21 (±0.03) | 0.20 (±0.04) | 0.24 (±0.05) | *P* = 0.18 |
| LVDD [cm] | 0.53 (±0.11) | 0.62 (±0.11) | 0.52 (±0.16) | *P* = 0.26 |
| PWD [cm] | 0.24 (±0.03) | 0.24 (±0.03) | 0.27 (±0.03) | *P* = 0.05 |
| IVSS [cm] | 0.29 (±0.04) | 0.26 (±0.05) | 0.32 (±0.06) | *P* = 0.09 |
| LVDS [cm] | 0.34 (±0.08) | 0.39 (±0.11) | 0.34 (±0.11) | *P* = 0.49 |
| PWS [cm] | 0.32 (±0.03) | 0.32 (±0.04) | 0.35 (±0.03) | *P* = 0.06 |
| EF [%] | 71.11 (±7.37) | 71.44 (±12.61) | 68.57 (±9.98) | *P* = 0.81 |
| FS [%] | 35.33 (±5.61) | 38.22 (±9.08) | 34.16 (±8.67) | *P* = 0.54 |
| LVEDV [ml] | 0.39 (±0.22) | 0.54 (±0.27) | 0.40 (±0.26) | *P* = 0.40 |
| LVESV [ml] | 0.11 (±0.07) | 0.18 (±0.13) | 0.13 (±0.08) | *P* = 0.27 |
| SV [ml] | 0.28 (±0.16) | 0.42 (±0.18) | 0.28 (±0.20) | *P* = 0.29 |
| AO [cm] | 0.40 (±0.05) | 0.42 (±0.04) | 0.38 (±0.06) | *P* = 0.39 |
| LA [cm] | 0.42 (±0.05) | 0.45 (±0.06) | 0.45 (±0.05) | *P* = 0.48 |
| CO [ml/min] | 87.54 (±64.27) | 132.45 (±45.57) | 92.49 (±69.45) | *P = 0.28* |

Abbreviations: IVSD, interventricular septum thickness at diastole; LVDD, left ventricular diastolic diameter; PWD, posterior wall thickness at diastole; IVSS, interventricular septum thickness at systole; LVDS, left ventricular diastolic diameter; PWS, posterior wall thickness at systole; EF, ejection fraction; FS, fractional shortening, LVEDV, left ventricular end-diastolic volume; LVESV, left ventricular end-systolic volume; AO, aortic root diameter; LA, left atrium diameter; CO; cardiac output; L group – TMA low-dose group; H group – TMA high-dose group. All data are expressed as the mean ± SD; ANOVA followed by post hoc Tuckey-test.
