# Supplementary material for: Trimethylamine, a gut bacteria metabolite and air pollutant, increases blood pressure and markers of kidney damage including proteinuria and KIM-1 in rats
Source: J Transl Med. 2022 Oct 15;20:470. doi: 10.1186/s12967-022-03687-y (PMC9571686; doi:10.1186/s12967-022-03687-y)
Supplement: Supplementary file 4 — Additional file 4: Table S3. Serum biochemical analysis. L group – TMA low-dose group; H group – TMA high-dose group. All data are expressed as the mean ± SD [file 12967_2022_3687_MOESM4_ESM.docx]

Additional Table 3. Serum biochemical analysis

| **Parameter** | **Control group** | **L group** | **H group** | **One-way ANOVA** |
| --- | --- | --- | --- | --- |
| Urea [mg/dl] | 91.56 (±12.55) | 79.86 (±17.09) | 87.00 (±15.87) | *P* = 0.33 |
| Creatinine [mg/dl] | 0.86 (±0.27) | 0.93 (±0.31) | 0.88 (±0.19) | *P* = 0.84 |
| Sodium [mg/dl] | 312.33 (±14.41) | 318.43 (±16.47) | 325.44 (±23.74) | *P* = 0.35 |
| Potassium [mg/dl] | 18.80 (±1.69) | 21.66 (±2.58) | 20.70 (±2.62) | *P* = 0.06 |
| Creatinine clearance [ml/min] | 0.92 (±0.73) | 1.30 (±0.77) | 0.99 (±0.42) | *P* = 0.49 |
| KIM-1 [pg/ml] | 436.72 (±78.79) | 388.17 (±42.29) | 405.54 (±59.65) | *P* = 0.32 |

Abbreviations: L group – TMA low-dose group; H group – TMA high-dose group. All data are expressed as the mean ± SD.
