# Supplementary figures and images for: Trimethylamine, a gut bacteria metabolite and air pollutant, increases blood pressure and markers of kidney damage including proteinuria and KIM-1 in rats
Source: J Transl Med. 2022 Oct 15;20:470. doi: 10.1186/s12967-022-03687-y (PMC9571686; doi:10.1186/s12967-022-03687-y)

## Slide 1
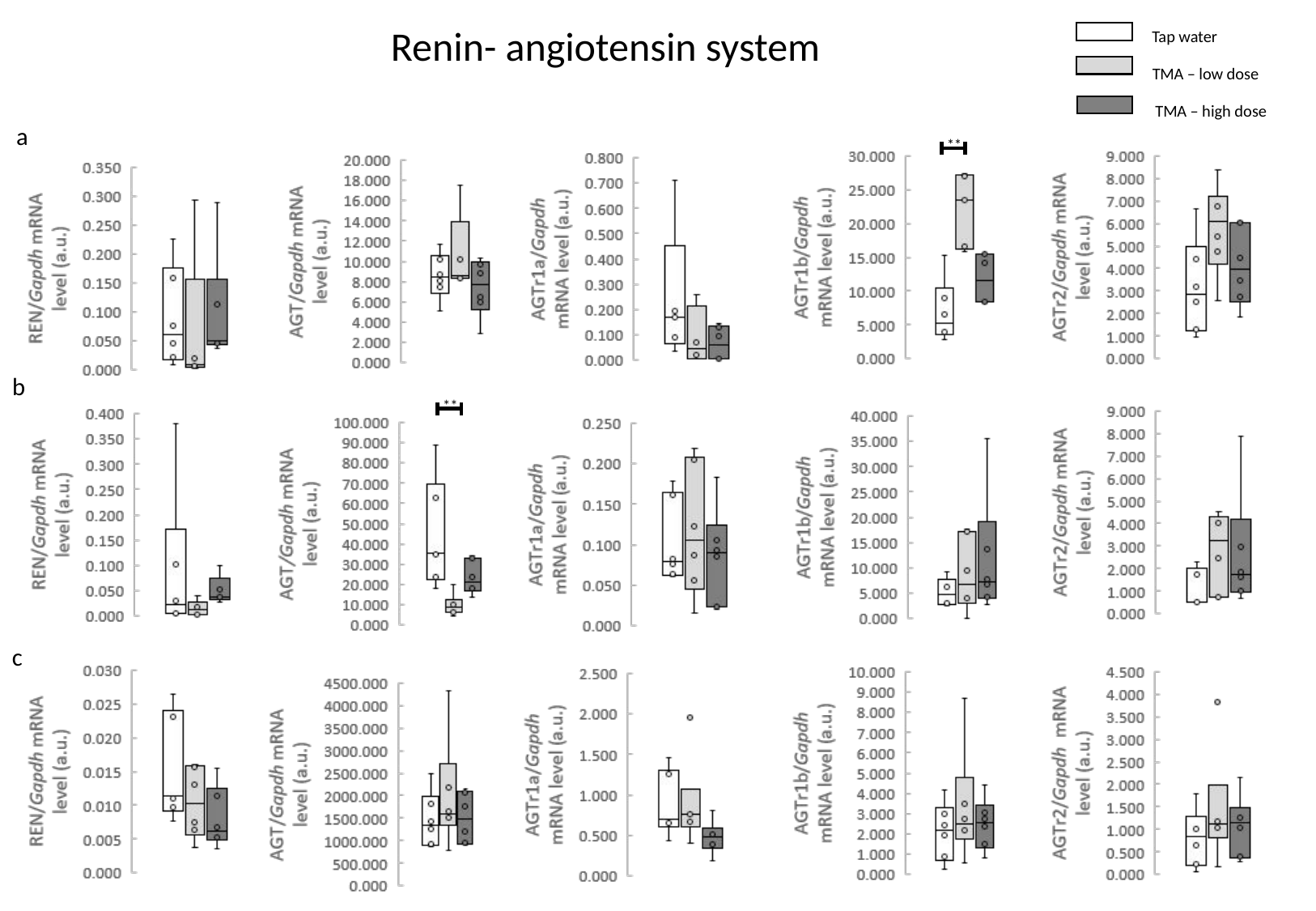

Renin- angiotensin system
Tap water
TMA – low dose
TMA – high dose
a
**
b
**
c

Supplement: Supplementary file 5 — Additional file 5: Fig. S1. Renin – angiotensin system. The genes of the renin-angiotensin system. RT-qPCR analysis of REN, AGT, AGTr1a, AGTr1b, AGTr2 transcript levels in the (A) renal cortex, (B) renal medulla, (C) liver in rats maintained either on tap water (control group) or low and high dose of TMA. Abbreviations: TMA, trimethylamine; REN, renin; AGT, angiotensinogen; AGTr1a, angiotensin II receptor, type 1a; AGTr1b, angiotensin II receptor, type 1b; AGTr2, angiotensin II receptor, type 2. All data are expressed as the median, Q1, Q3, MIN, MAX (n = 6; use arbitrary units); Kruskal–Wallis test followed by post-hoc Dunn’s test. **P < 0.01 vs control group [file 12967_2022_3687_MOESM5_ESM.pptx]

## Slide 1
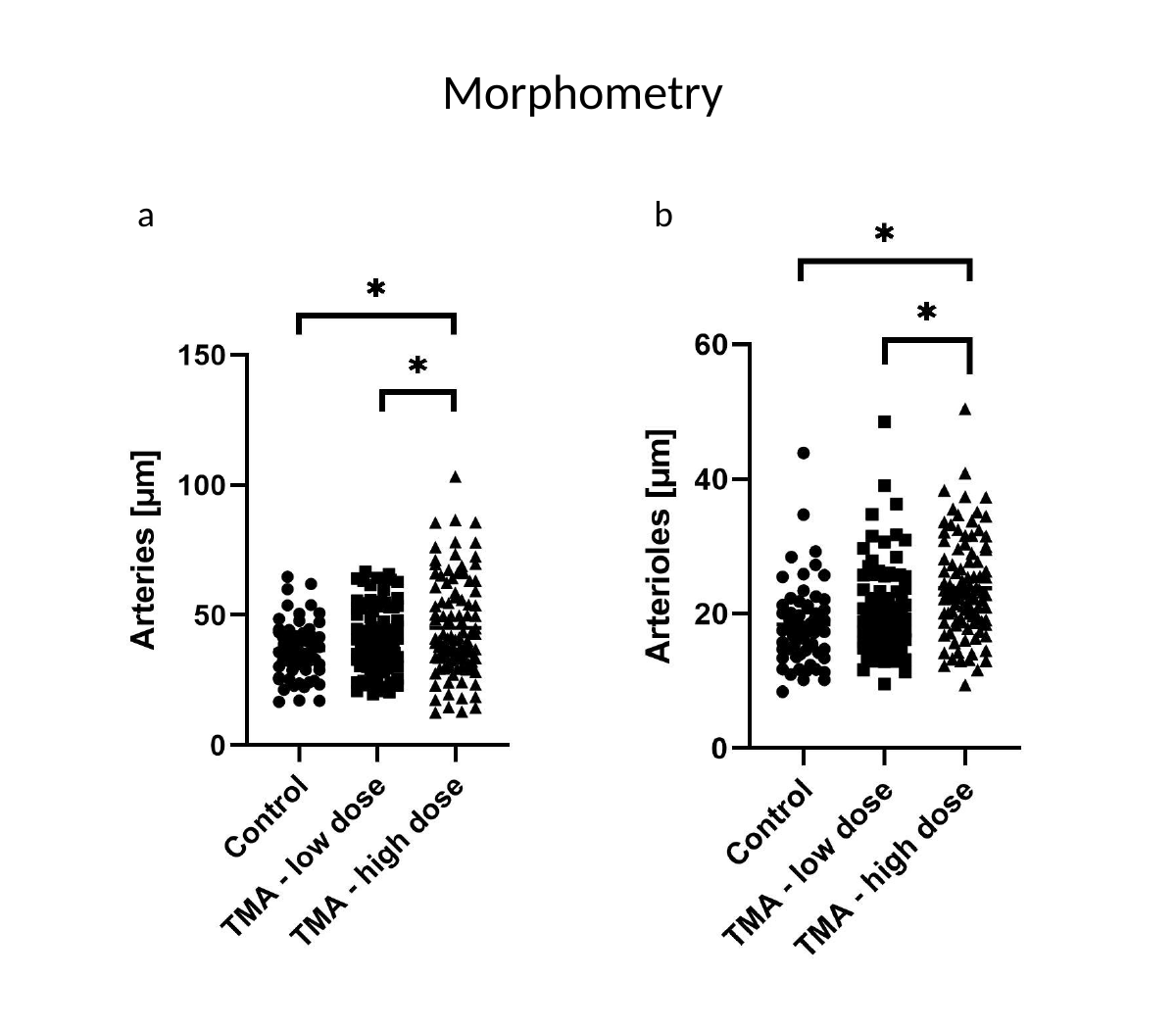

Morphometry
a
b

Supplement: Supplementary file 6 — Additional file 6: Fig. S2. Morphometry. Morphometric measurement of renal (a) arteries and (b) arterioles in rats maintained either on tap water (control group) or low and high dose of TMA. Abbreviations: TMA, trimethylamine. Morphometric measurements of five arcuate arteries and five arterioles were performed for each individual. Four measurements were made for each vessel. All measurements are expressed as single points (n = 3–5); ANOVA followed by post-hoc Tuckey test; *P < 0.05 [file 12967_2022_3687_MOESM6_ESM.pptx]
